# Supplementary material for: IRX2 regulates angiotensin II-induced cardiac fibrosis by transcriptionally activating EGR1 in male mice
Source: Nat Commun. 2023 Aug 16;14:4967. doi: 10.1038/s41467-023-40639-6 (PMC10432509; doi:10.1038/s41467-023-40639-6)
Supplement: Supplementary file 1 — Supplementary Information [file 41467_2023_40639_MOESM1_ESM.pdf]

Supplementary material

IRX2 regulates angiotensin II-induced cardiac fibrosis by transcriptionally activating EGR1 in mice

Authors: Zhen-Guo Ma[1,2,3]; Yu-Pei Yuan[1,2,3]; Di Fan[1,2,3]; Xin Zhang[1,2,3]; Teng Teng[1,2,3]; Peng Song[1,2,3]; Chun-Yan Kong[1,2,3]; Can Hu[1,2,3]; Wen-Ying Wei[1,2,3]; Qi-Zhu Tang[1,2,3]

[1]Department of Cardiology, Renmin Hospital of Wuhan University, 430060 Wuhan, PR China
[2]Cardiovascular Research Institute of Wuhan University, 430060 Wuhan, PR China
[3]Hubei Key Laboratory of Metabolic and Chronic Diseases, 430060 Wuhan, PR China

Address for Correspondence:
Qi-Zhu Tang, PhD
Department of Cardiology,
Renmin Hospital of Wuhan University, 430060 Wuhan, PR China
Cardiovascular Research Institute of Wuhan University, 430060 Wuhan, PR China
Hubei Key Laboratory of Metabolic and Chronic Diseases, 430060 Wuhan, PR China.
E-mail: qztang@whu.edu.cn

Figure S1

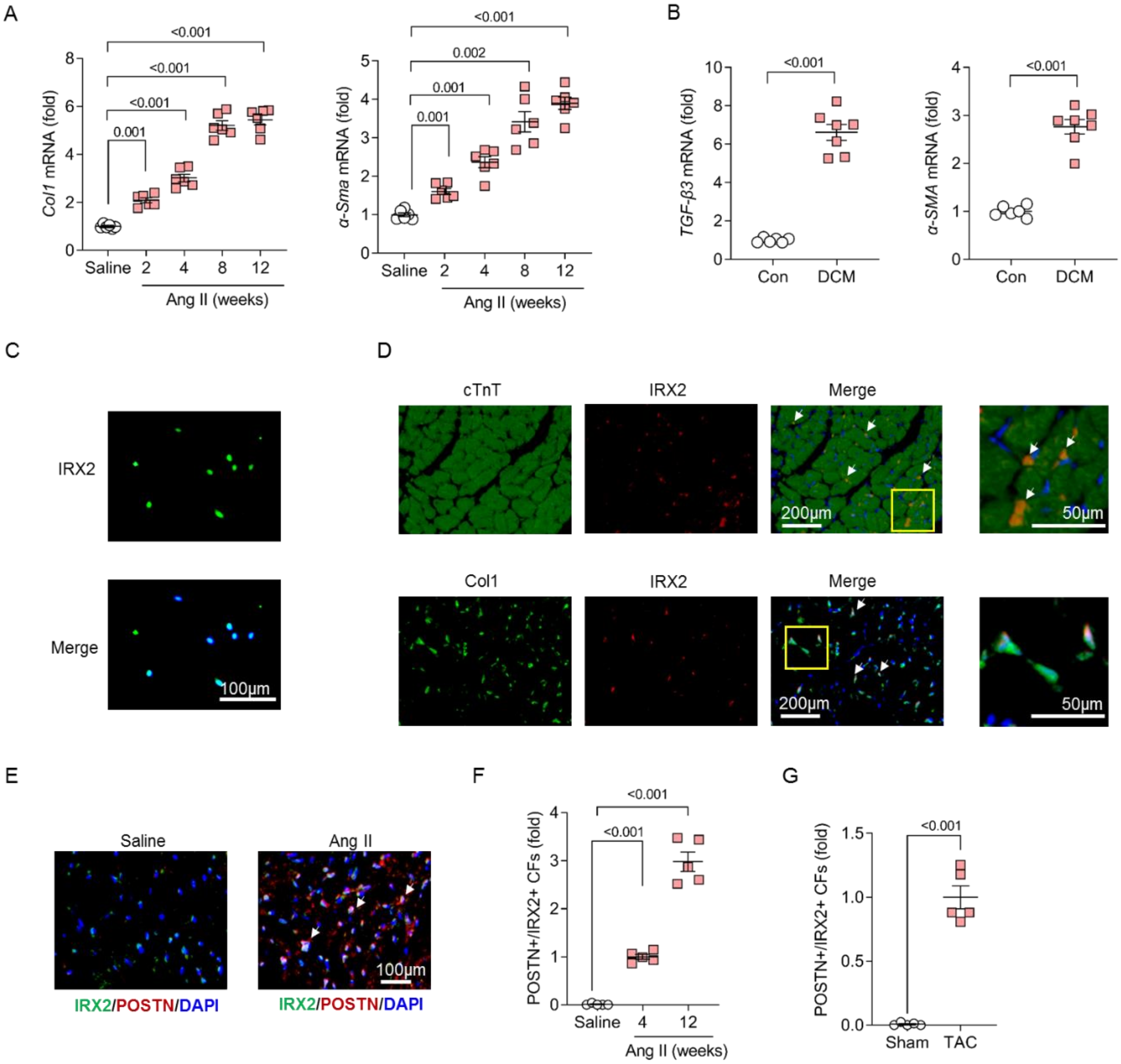

Supplementary Figure 1 IRX2 was mainly expressed in adult cardiac fibroblasts. A, Relative mRNA levels of *Col1* and *α-Sma* in the heart after Ang II infusion by an osmotic minipump (*n*=6). **B**, Relative mRNA levels of *TGF-β3* and *α-SMA* in heart samples obtained from patients with dilated cardiomyopathy (DCM) and control (Con) donors (Con, *n*=6; DCM, *n*=7). **C**, Representative images of IRX2 (green) staining in CFs isolated from wild-type mice. Nuclei were stained with DAPI (blue) (*n*=4). **D**, Representative images of IRX2 (red), cardiac Troponin T (cTnI, green, top), and Col1 (green, bottom) in heart samples. Nuclei were stained with DAPI (blue). Hearts were subjected to immunofluorescence staining to detect the cellular location of IRX2 (*n*=4). **E,** Representative images of IRX2 (green), periostin (POSTN, red) in 12 week-Ang II-infused heart samples. Nuclei were stained with DAPI (blue). Hearts were subjected to immunofluorescence staining to detect POSTN+/IRX2+ CFs (*n*=5). **F-G**, Statistical analysis of POSTN+/IRX2+ CFs in Ang II or transverse aortic constriction (TAC)-treated hearts (*n*=5). Data are shown as the mean ± SEM, and analysed using one-way

ANOVA followed by Tukey post hoc test (**F**) or Tamhane's T2 test (**A**). For the analysis in (**B** and **G**), an unpaired two-tailed Student′s *t* test was conducted. Source data are provided as a Source Data file

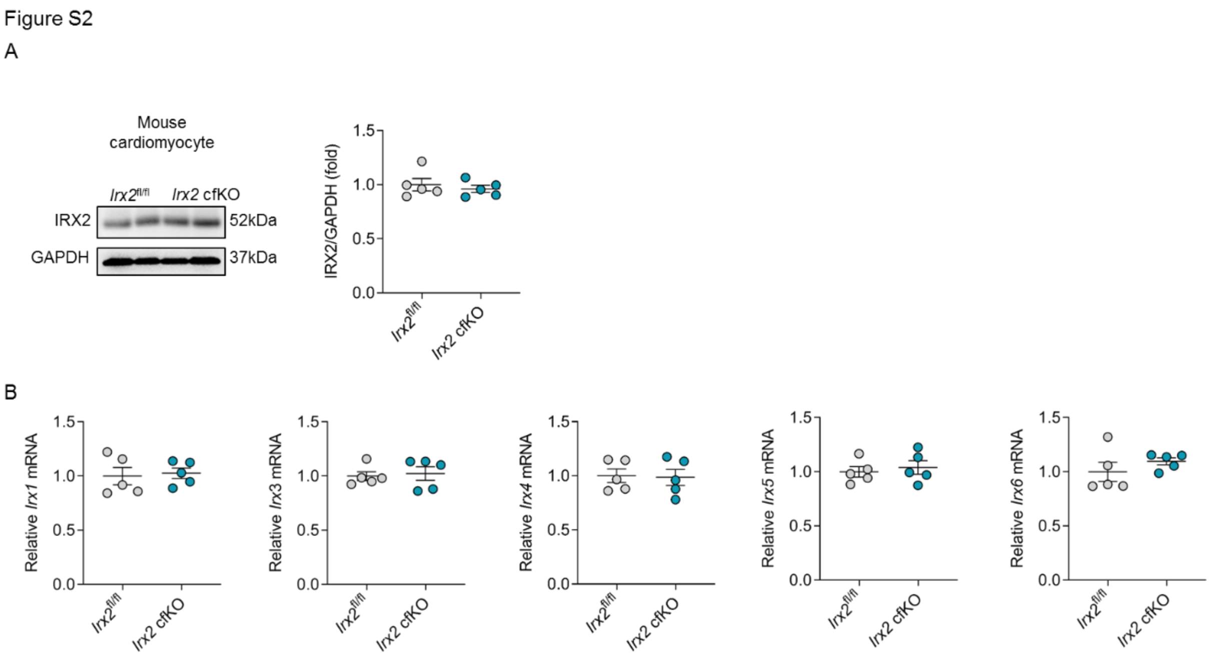

Supplementary Figure 2 Conditional fibroblast-specific *Irx2*-depleted mice were generated to investigate the role of IRX2 in cardiac fibrosis induced by angiotensin II (Ang II) infusion. A, Representative western blots and statistical analysis of IRX2 protein expression in cardiomyocytes isolated from *Irx2* cfKO mice and littermate controls (*n*=5). **B**, The mRNA levels of other *Irx* members in *Irx2*-deficient CFs (*n*=5). Data are shown as the mean ± SEM, and analysed using an unpaired two-tailed Student′s *t* test. Source data are provided as a Source Data file.

Figure S3

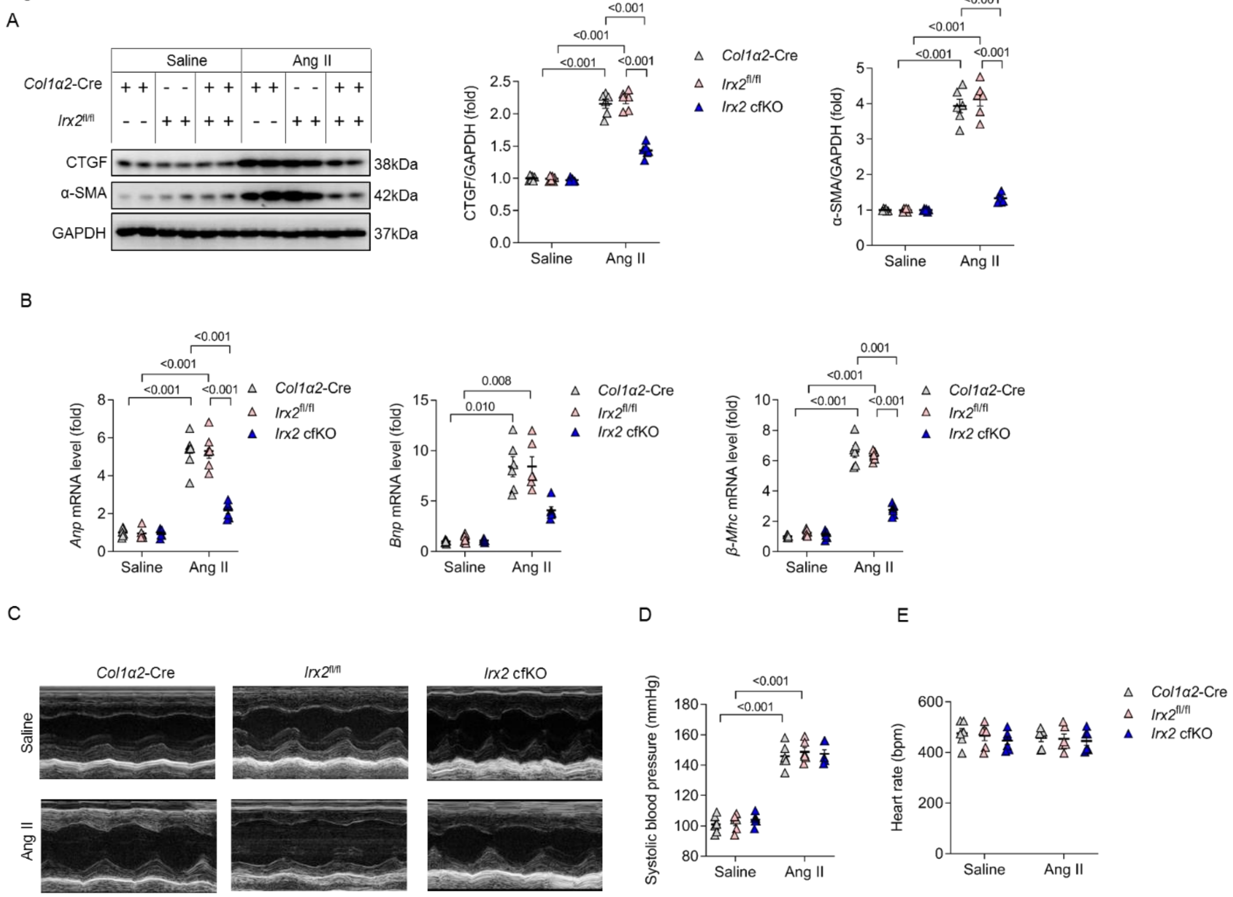

Supplementary Figure 3 Conditional fibroblast-specific *Irx2* depletion attenuated angiotensin II (Ang II) infusion-induced cardiac fibrosis. A, Representative western blots and statistical analysis of α-smooth muscle actin (α-SMA) and connective tissue growth factor (CTGF) expression in hearts from *Irx2* cfKO mice and littermate controls after Ang II infusion (*n*=6). **B**, Relative mRNA levels of *Anp*, *Bnp*, and *β-Mhc* detected in hearts from *Irx2* cfKO mice and control mice (*n*=6). **C**, Representative echocardiograms in the indicated groups. **D**, Systolic blood pressure of *Irx2* cfKO and littermate controls (*n*=6). **E**, Heart rate of *Irx2* cfKO and littermate controls (*n*=6). Data are shown as the mean ± SEM, and analysed using one-way ANOVA followed by Tukey post hoc test (**D-E**) or Tamhane's T2 test (**A-B**). Source data are provided as a Source Data file.

Figure S4

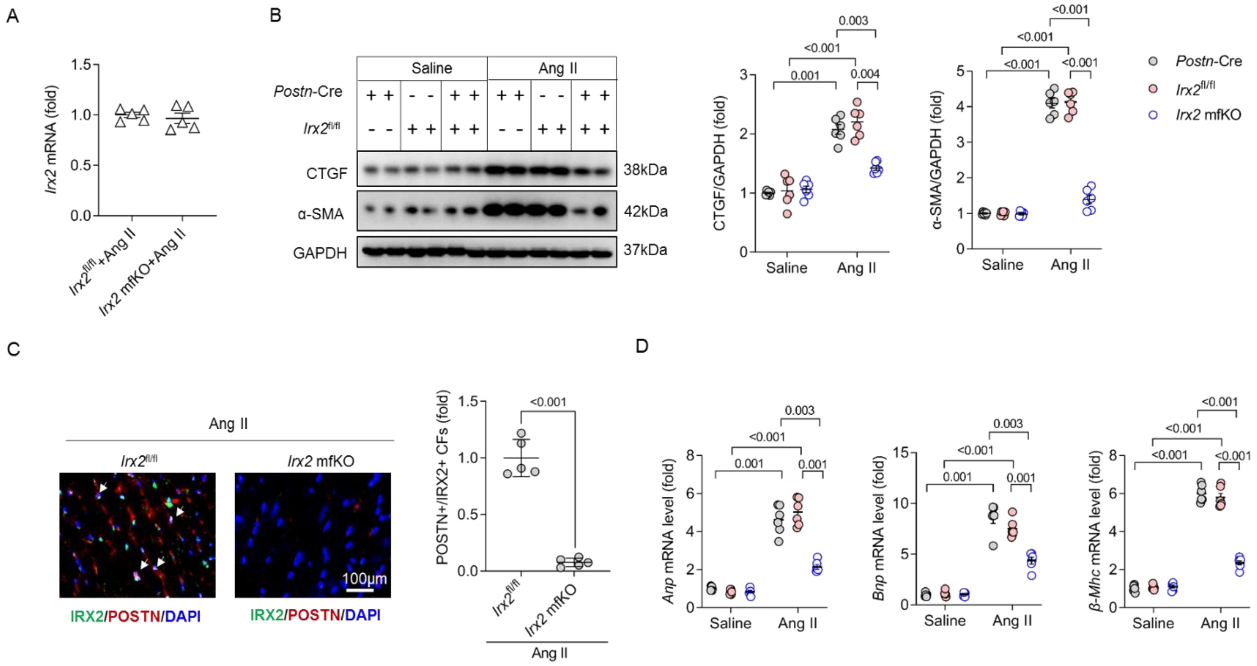

Supplementary Figure 4 Conditional myofibroblast-specific *Irx2* depletion attenuated angiotensin II (Ang II)-induced fibrotic remodelling in mice. A, *Irx2* mRNA expression in cardiomyocytes isolated from *Irx2* mfKO mice and littermate controls after Ang II infusion for 12 weeks (*n*=5). **B**, Representative western blots and statistical analysis of α-smooth muscle actin (α-SMA) and connective tissue growth factor (CTGF) in hearts from *Irx2* mfKO mice and control mice (*n*=6). **C**, Representative images and statistical analysis of POSTN+/IRX2+ CFs in hearts (*n*=5). **D**, Relative mRNA levels of *Anp*, *Bnp*, and *β-Mhc* detected in hearts from *Irx2* mfKO mice and control mice after Ang II infusion (*n*=6). Data are shown as the mean ± SEM, and analysed using one-way ANOVA followed by Tamhane's T2 test (**B** and **D**). For the analysis in (**A** and **C**), an unpaired two-tailed Student′s *t* test was conducted. Source data are provided as a Source Data file.

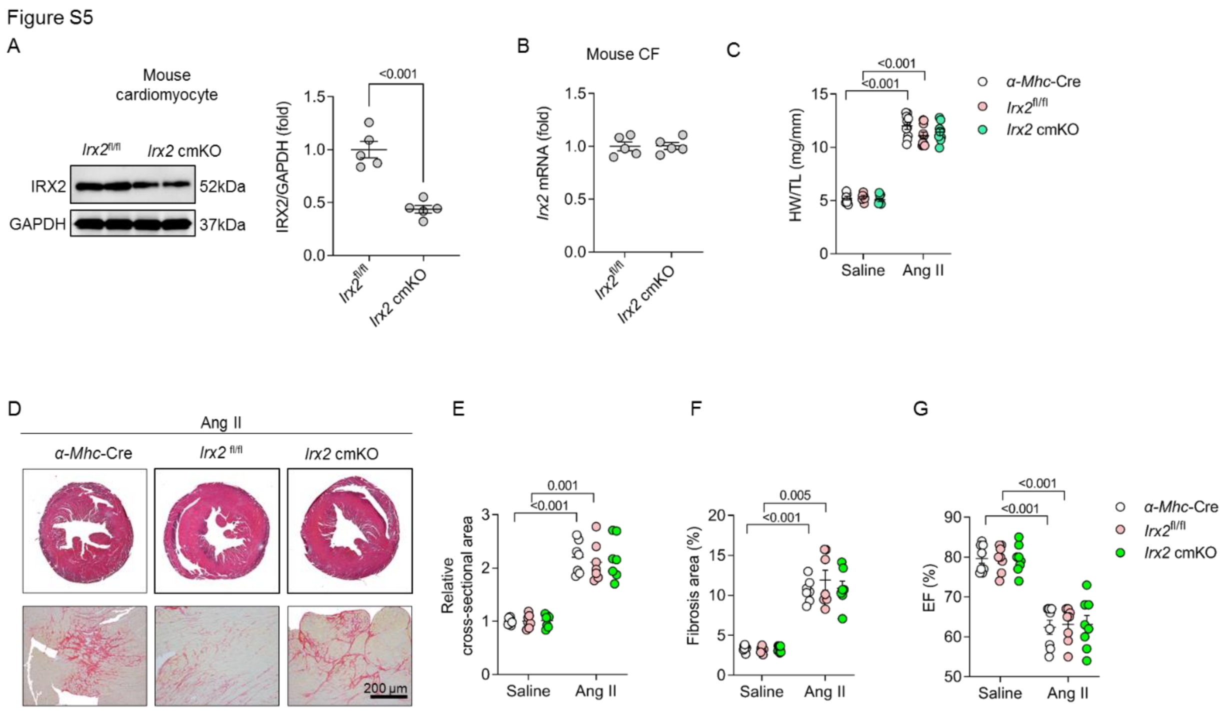

Supplementary Figure 5 Conditional cardiomyocyte-specific *Irx2* depletion did not affect angiotensin II (Ang II)-induced fibrotic remodelling in mice. Conditional cardiomyocyte-specific *Irx2*-deficient mice (*Irx2* cmKO) were bred by crossing mice with a conditional knockout allele of *Irx2* (*Irx2*$^{fl/fl}$) with *α-Mhc*-Cre mice. *Irx2* cmKO mice and littermate controls were subjected to Ang II infusion for 12 weeks. **A**, Representative western blots and statistical analysis of IRX2 protein expression in cardiomyocytes isolated from *Irx2* cmKO mice and littermate controls (*n*=5). **B**, *Irx2* mRNA in CFs isolated from *Irx2* cmKO mice and littermate controls after Ang II infusion for 12 weeks (*n*=5). **C**, Heart weight-to-tibia length (HW/TL) ratio (*n* = 8 mice, *α-Mhc*-Cre+Saline; *n* = 8 mice, *Irx2*$^{fl/fl}$+Saline; *n* = 8 mice, *Irx2* cmKO+Saline; *n* = 10 mice, *α-Mhc*-Cre+TAC; *n* = 10 mice, *Irx2*$^{fl/fl}$+TAC; *n* = 9 mice, *Irx2* cmKO+TAC). **D**, Histological staining showed heart enlargement and cardiac fibrosis after Ang II infusion. **E**, The cell area of cardiomyocytes was determined after Ang II infusion (*n*=7). **F**, Cardiac fibrosis was determined by picrosirius red staining of samples from *Irx2* cmKO mice and littermate controls after Ang II infusion (*n*=7). **G**, The ejection fraction (EF) was detected in these groups (*n*=8). Data are shown as the mean ± SEM, and analysed using one-way ANOVA followed by Tukey post hoc test (**G**) or Tamhane's T2 test (**C, E, F**). For the analysis in (**A-B**), an unpaired two-tailed Student′s *t* test was conducted. Source data are provided as a Source Data file.

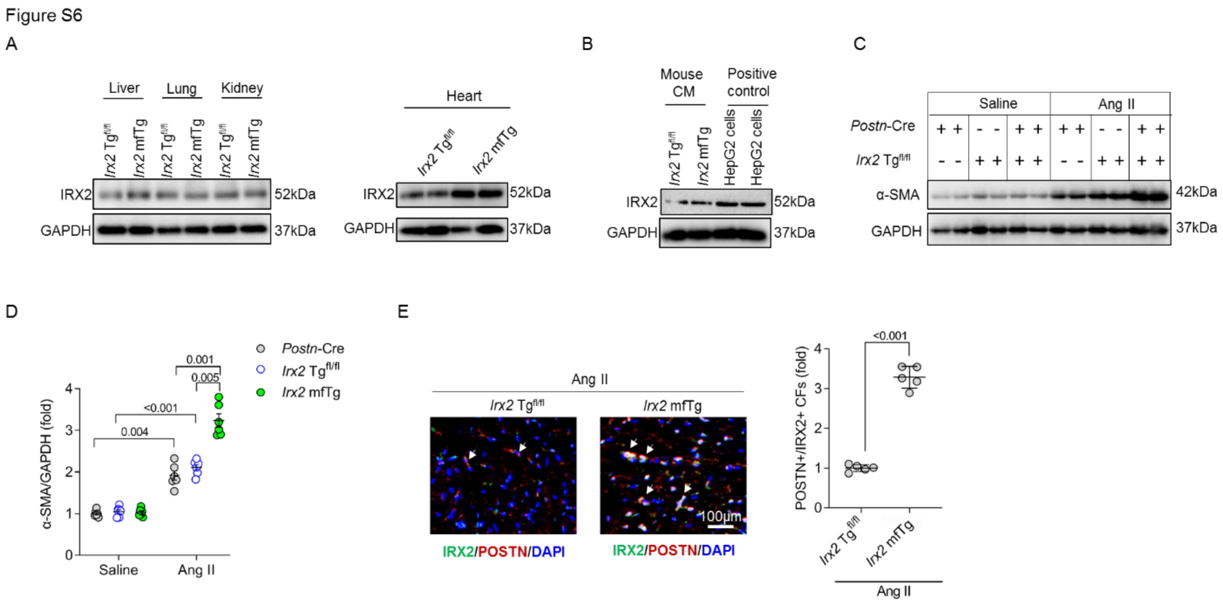

Supplementary Figure 6 Conditional myofibroblast-specific *Irx2*-overexpressing mice were generated. Conditional myofibroblast-specific *Irx2*-overexpressing mice (*Irx2* mfTg) were bred by crossing mice with a conditional transgenic allele of *Irx2* (*Irx2* Tg$^{fl/fl}$) with *Postn*-Cre mice. *Irx2* mfTg mice and littermate controls were subjected to angiotensin II (Ang II) infusion for 4 weeks. **A**, Representative western blots of IRX2 protein expression in the liver, lungs, kidneys and heart of *Irx2* mfTg mice and littermate controls after Ang II infusion (*n*=4). **B**, Representative western blots of IRX2 protein expression in cardiomyocytes (CMs) isolated from *Irx2* mfTg mice and littermate controls after Ang II infusion for 4 weeks (*n*=5). HepG2 cells were used as the positive control. **C-D**, Representative western blots and statistical analysis of the protein α-smooth muscle actin (α-SMA) in hearts from *Irx2* mfTg mice and littermate controls after Ang II infusion (*n*=6). **E**, Representative images and statistical analysis of POSTN+/IRX2+ CFs in hearts (*n*=5). Data are shown as the mean ± SEM, and analysed using one-way ANOVA followed by Tamhane's T2 test (**D**). For the analysis in (**E**), an unpaired two-tailed Student′s *t* test was conducted. Source Data are provided as a Source Data file.

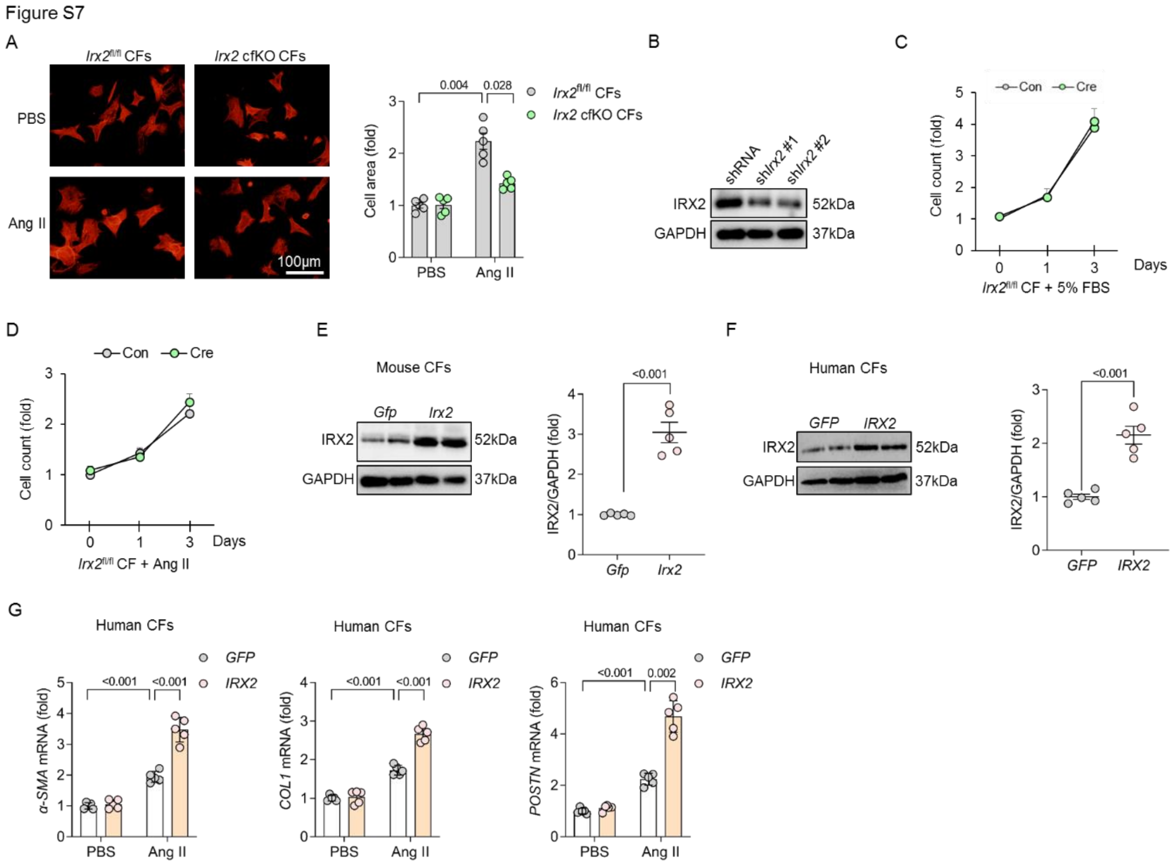

Supplementary Figure 7 IRX2 did not affect adult cardiac fibroblast (CF) proliferation in vitro. **A**, CFs were isolated from *Irx2* cfKO mice and *Irx2*$^{fl/fl}$ mice and then stimulated with angiotensin II (Ang II) for another 24 h to induce a fibrotic phenotype. Then, conditioned medium was collected from these myofibroblasts and used to treat neonatal rat cardiomyocytes in the absence or presence of Ang II for 24 h. After that, the cell area of cardiomyocytes was detected by immunofluorescence staining with anti-α-actinin (red) (*n*=5). **B**, Representative western blots of IRX2 protein expression in CFs transfected with shRNA. **C**, The cell counts of CFs with *Irx2* depletion were measured after incubation with 5% FBS (*n*=3 for each time point). **D**, The cell counts of CFs with *Irx2* depletion were measured after incubation with Ang II (*n*=4 for each time point). **E**, Representative western blots and statistical analysis of IRX2 protein expression in mouse CFs infected with an adenovirus carrying *Irx2* (*n*=5). **F**, Representative western blots and statistical analysis of IRX2 protein expression in human CFs infected with an adenovirus carrying *IRX2* (*n*=5). **G**, Human CFs were infected with an adenoviral vector to overexpress *IRX2* and then subjected to Ang II treatment for 24 h. Total RNA was extracted to detect the mRNA levels of *α-SMA*, *COL1* and *POSTN* (*n*=5). Data are shown as the mean ± SEM, and analysed using one-way ANOVA followed by Tamhane's T2 test (**A, G**). For the analysis in (**E-F**), an unpaired two-tailed Student′s *t* test was conducted. For the analysis in (**C-D**), repeated measures ANOVA was conducted. Source data are provided as a Source Data file.

Figure S8

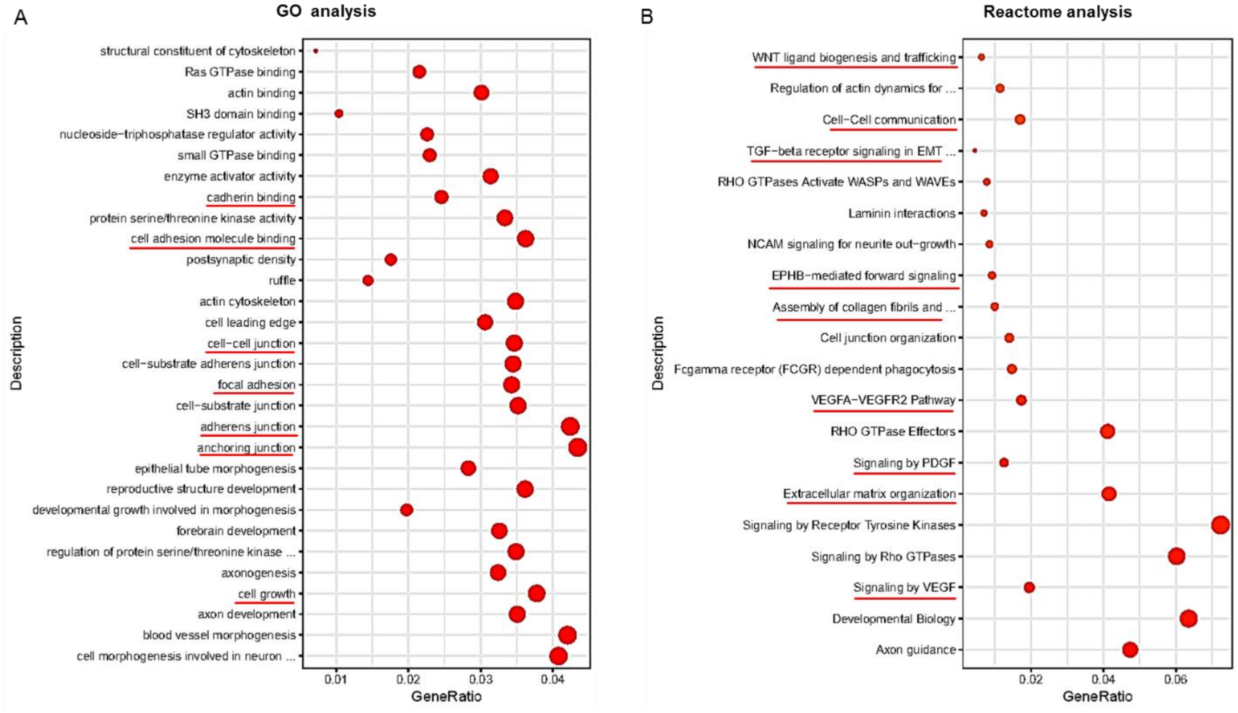

Supplementary Figure 8 GO pathway and Reactome pathway analyses of the RNA-sequencing dataset of *Irx2*-overexpressing cardiac fibroblasts. A, GO pathway analysis based on differentially expressed genes identified from the RNA-sequencing dataset of *Irx2*-overexpressing cardiac fibroblasts. **B**, Reactome pathway analysis was performed based on the RNA-sequencing dataset of *Irx2*-overexpressing cardiac fibroblasts. Several signalling pathways, such as the Wnt signalling pathway, the TGF-β receptor signalling, the VEGF signalling, the PDGF signalling, the assembly of collagen fibrils and extracellular matrix organization, were found to be regulated by IRX2. Only significant pathways (padj<0.01) are shown.

Figure S9

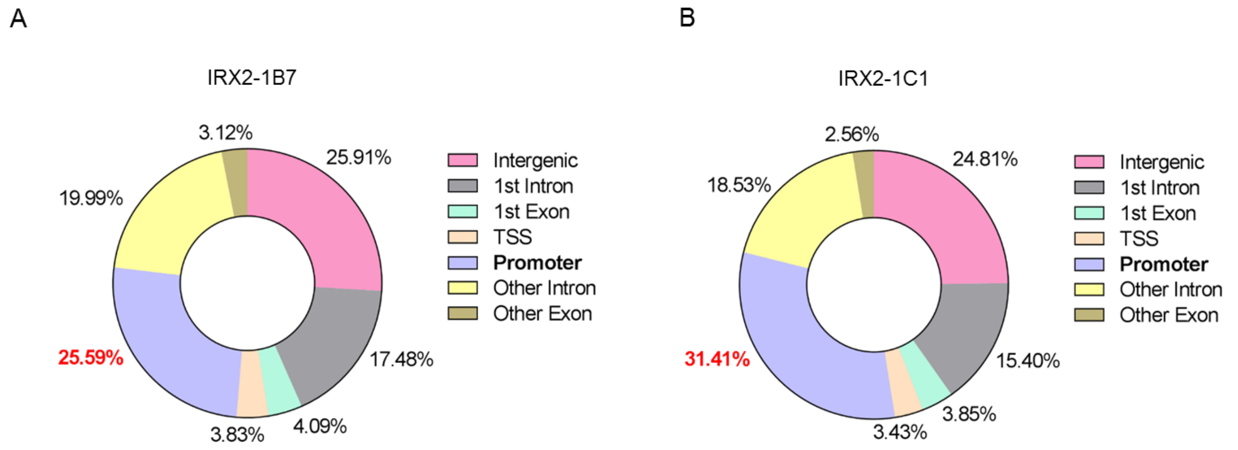

Supplementary Figure 9 Pie chart of the distribution of the IRX2 binding sites identified by ChIP-Seq. A, Pie chart of the distribution of the IRX2 binding sites identified by ChIP-Seq with the IRX2-1B7 antibody. **B**, Pie chart of the distribution of the IRX2 binding sites identified by ChIP-Seq with the IRX2-1C1 antibody.

Figure S10

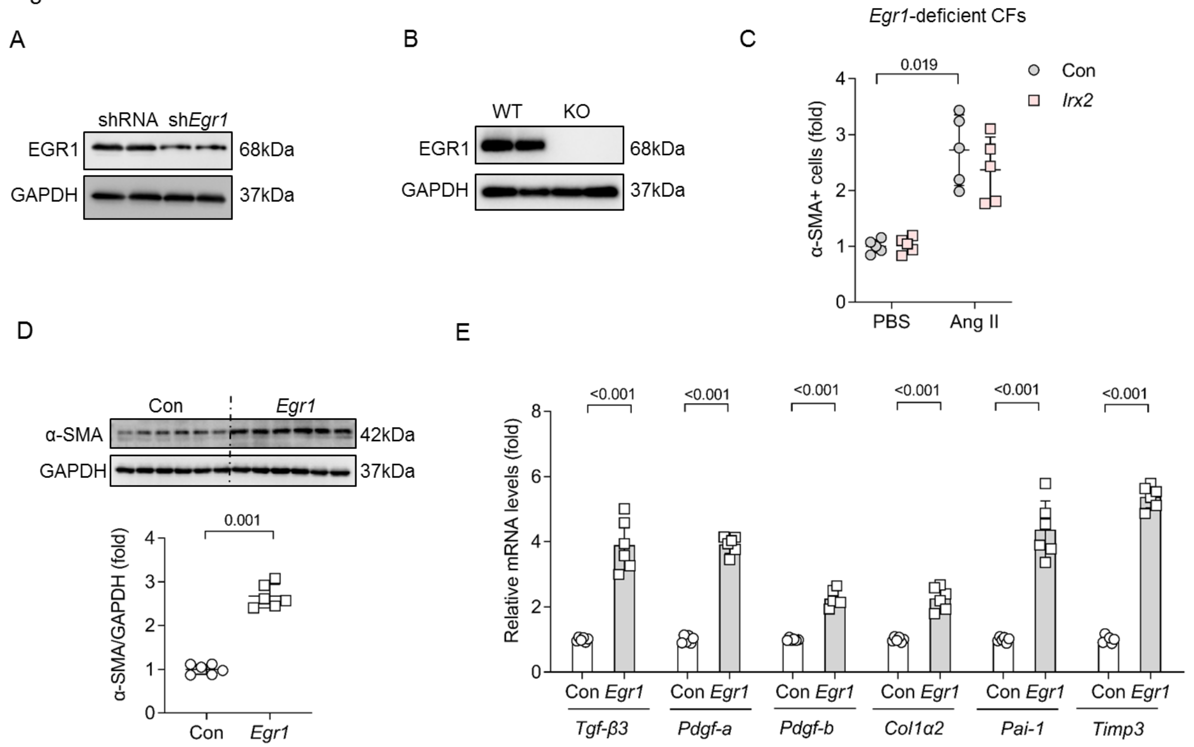

Supplementary Figure 10 EGR1 mediated the fibrotic response in *Irx2*-overexpressing cardiac fibroblasts. **A**, Representative western blots of EGR1 protein expression in CFs transfected with shRNA (*n*=4). **B**, EGR1 protein expression was detected in CFs isolated from *Egr1*-deficient mice (*n*=4). **C**, Statistical analysis of α-smooth muscle actin (α-SMA)+ cells in response to Ang II treatment for 24 h (*n*=5, for each experiment, 50-60 fields were counted). **D**, Representative western blots and statistical analysis of α-SMA protein expression in *Egr1*-overexpressing CFs (*n*=6). **E**, The mRNA levels of *Tgf-β3*, *Pdgf-a*, *Pdgf-b*, *Col1α2, Pai-1* and *Timp3* in *Egr1*-overexpressing CFs (*n*=5). Data are shown as the mean ± SEM, and analysed using one-way ANOVA followed by Tukey test (**C**). For the analysis in (**D-E**), an unpaired two-tailed Student′s *t* test was conducted. Source data are provided as a Source Data file.

Figure S11

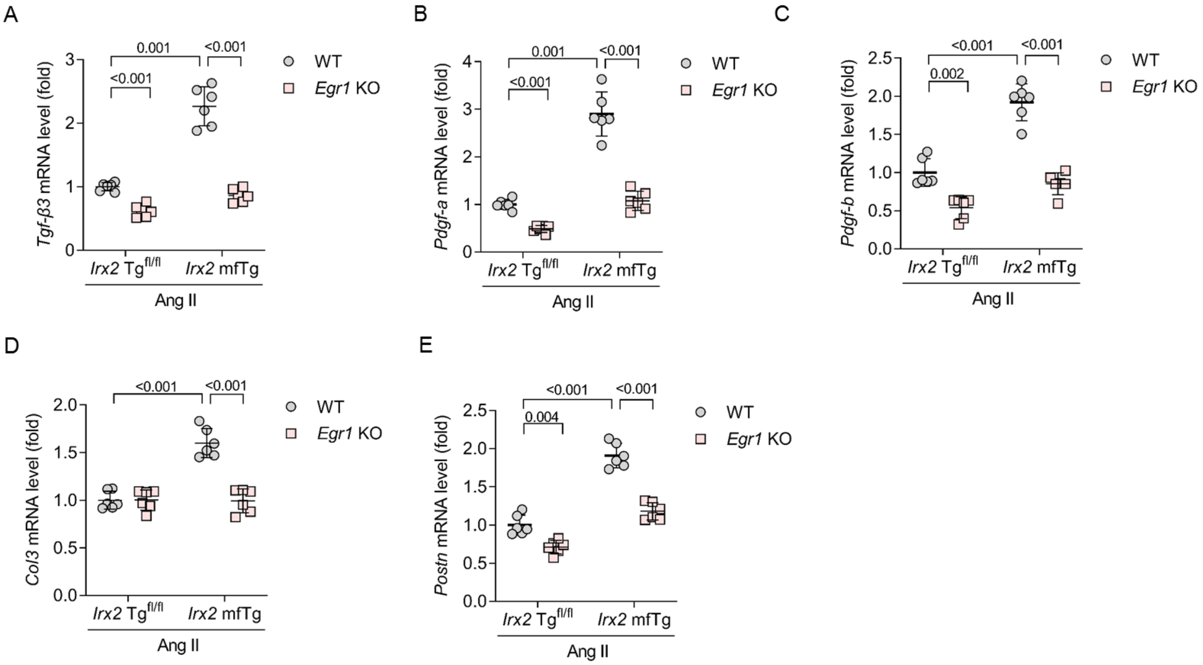

Supplementary Figure 11 Genetic depletion of *Egr1* decreased fibrotic markers expression in mice with conditional myofibroblast-specific *Irx2* overexpression after angiotensin II (Ang II) infusion for 4 weeks. Conditional myofibroblast-specific *Irx2*-overexpressing mice (*Irx2* mfTg) were bred with *Egr1* global knockout mice. The resulting mouse line and littermate controls were subjected to Ang II infusion for 4 weeks. **A-E**, Relative mRNA levels of *Tgf-β3* (**A**), *Pdgf-a* (**B**), *Pdgf*-b (**C**), *Col3* (**D**) and *Postn* (**E**) in the heart (n=6). Data are shown as the mean ± SEM, and analysed using one-way ANOVA followed by Tukey post hoc test (**C-E**) or Tamhane's T2 test (**A-B**). Source data are provided as a Source Data file.

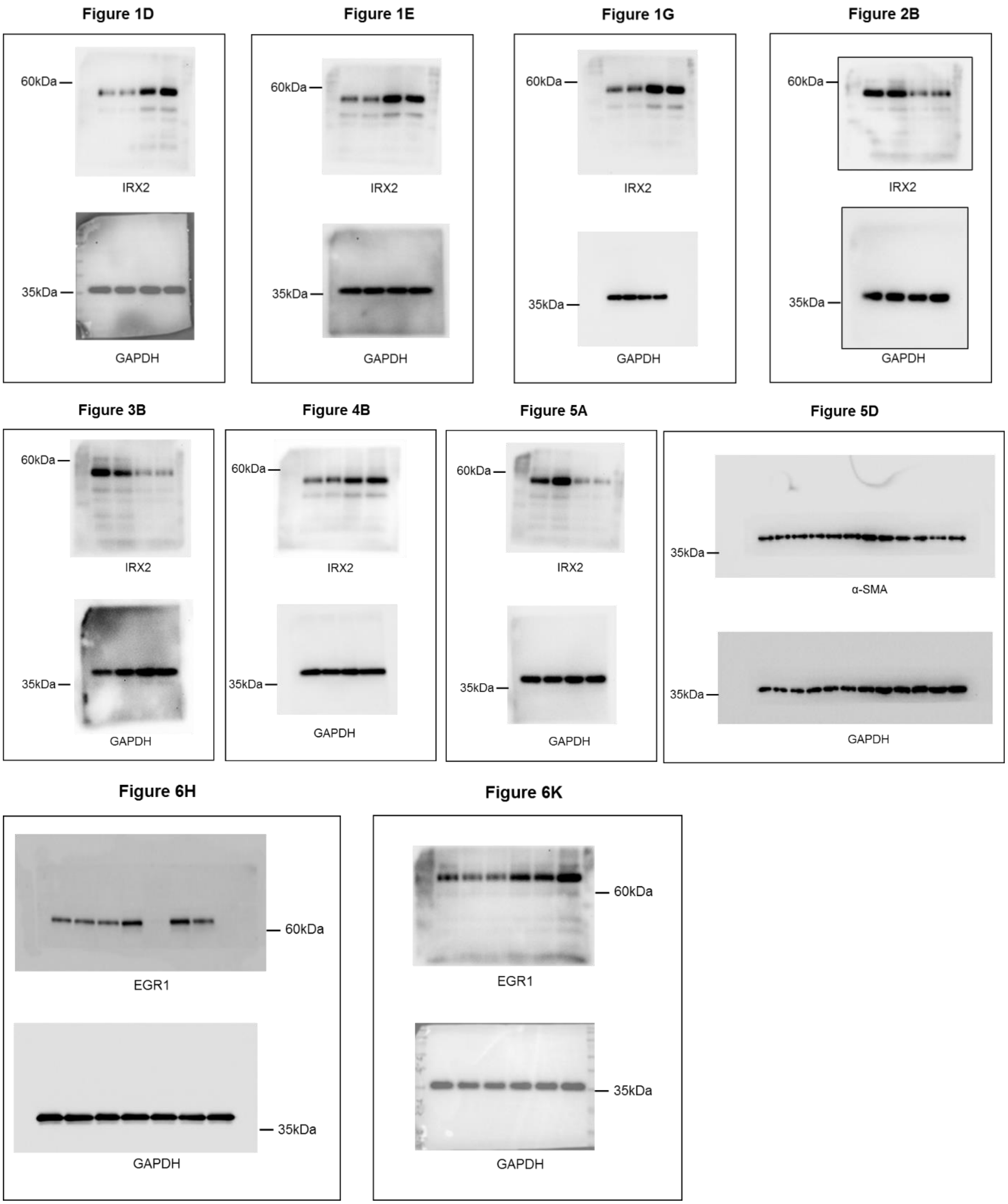

Supplementary Figure 12. Uncropped gels relating to indicated figures.

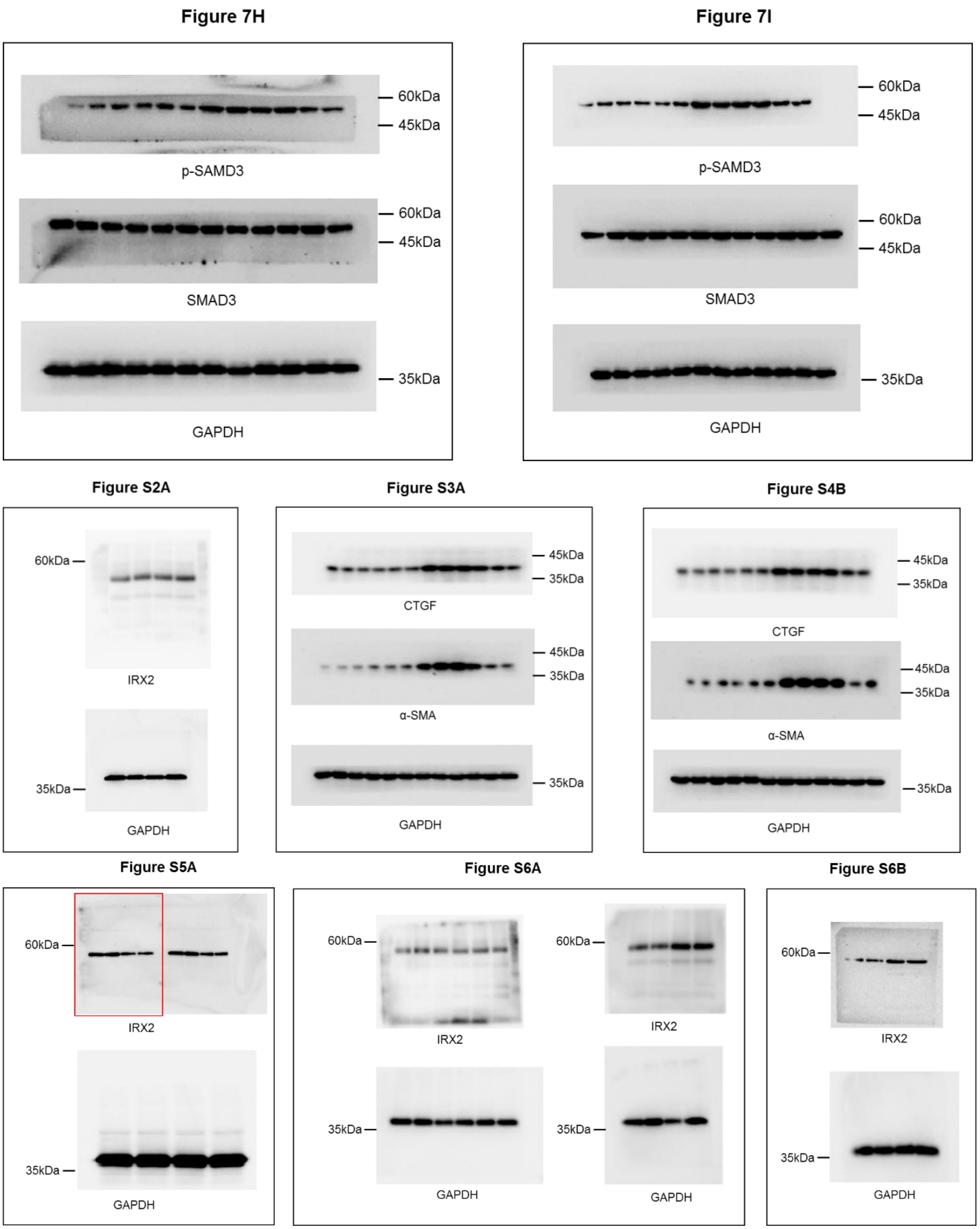

Supplementary Figure 12. Uncropped gels relating to indicated figures (continued).

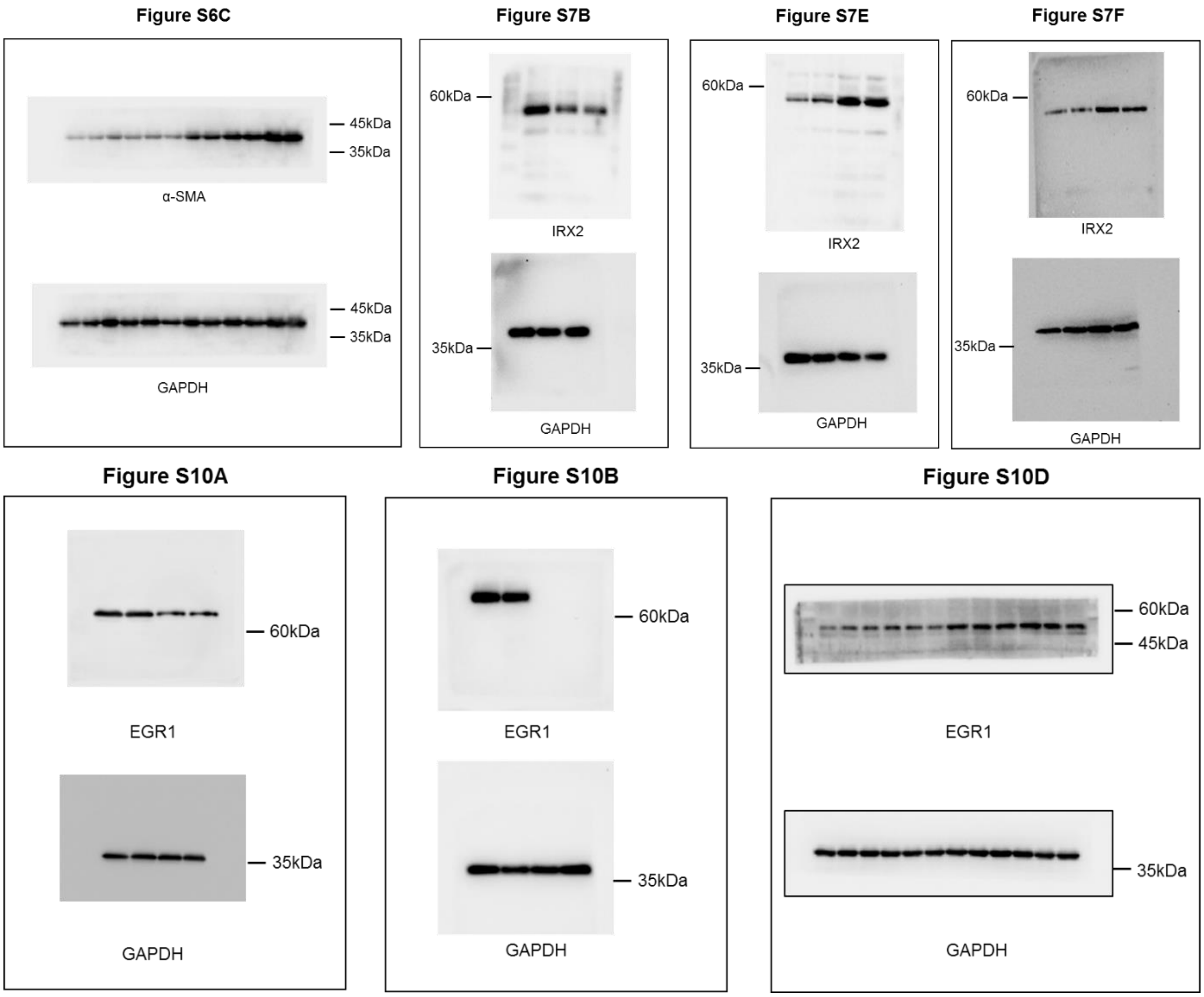

Supplementary Figure 12. Uncropped gels relating to indicated figures (continued).

Supplementary Table 1 Primer sequences used in our study

Gene	Species	Sequence	
GAPDH	Human	Forward	AGCCACATCGCTCAGACAC
		Reverse	GCCCAATACGACCAAATCC
IRX1	Human	Forward	CCAGTTCCAATACGGGGACC
		Reverse	CCATCGTGCTCGTCGATCTT
IRX2	Human	Forward	CCGAGAAACAAAAGCGAAGA
		Reverse	AGCACGAGTGATCCGTGAG
IRX3	Human	Forward	AAAAGTTACTCAAGACAGCTTTCCA
		Reverse	GGATGAGGAGAGAGCCGATA
IRX4	Human	Forward	ACTACCCTTACGAGCCAGC
		Reverse	CCTTGAGCGTGCTGGTGG
IRX5	Human	Forward	CCTATCCGCAGGGCTACTTG
		Reverse	CTCAGCTCCTCCTGCTTCG
IRX6	Human	Forward	CTCAACGAGCACCGCAAAAA
		Reverse	TCTCTGGGGACTGAGAGTCG
EGR1	Human	Forward	ACCTGACCGCAGAGTCTTTTC
		Reverse	GATGAGCTGGGACTGGTAGC
Irx1	Mouse	Forward	TTATCCCTATGGTCAGTTTCAATACG
		Reverse	CGTTGAGCCAGGCTTTCAG
Irx2	Mouse	Forward	ACGCACACCACCGGAATG
		Reverse	ATGGATAGGCCGCACTGC
Irx3	Mouse	Forward	CGCCTCAAGAAGGAGAACAAGA
		Reverse	CGCTCGCTCCCATAAGCAT
Irx4	Mouse	Forward	GCGGGCCGGCTCTTTCCTG
		Reverse	AGTTCTAGCTCCTTGTCGTCTTTG
Irx5	Mouse	Forward	GGCTACAACTCGCACCTCCA
		Reverse	CCAAGGAACCTGCCATACCG
Irx6	Mouse	Forward	GGCGGCCTGCTCCTGCAGCCC
		Reverse	GGATGTGCTGCCATACGGGTGT
Gapdh	Mouse	Forward	ACTCCACTCACGGCAAATTC
		Reverse	TCTCCATGGTGGTGAAGACA
Anp	Mouse	Forward	ACCTGCTAGACCACCTGGAG
		Reverse	CCTTGGCTGTTATCTTCGGTACCGG
Bnp	Mouse	Forward	GCTGCTTTGGGCACAAGATAG
		Reverse	GGTCTTCCTACAACAACTTCAG

β-Mhc	Mouse	Forward	CCGAGTCCCAGGTCAACAA
		Reverse	CTTCACGGGCACCCTTGGA
Col1	Mouse	Forward	CCCAACCCAGAGATCCCATT
		Reverse	GAAGCACAGGAGCAGGTGTAGA
Col3	Mouse	Forward	CCCAACCCAGAGATCCCATT
		Reverse	GAAGCACAGGAGCAGGTGTAGA
α-Sma	Mouse	Forward	TGGCTCTGTGCAAGAAGCGGAT
		Reverse	AATCTGGGCGTTGTCACTGCGT
Egr1	Mouse	Forward	CCTTTTCTGACATCGCTCTGAA
		Reverse	CGAGTCGTTTGGCTGGGATA
Tgf-β3	Mouse	Forward	ATGACCCACGTCCCCTATCA
		Reverse	ACTCAGACTCCGAGGTCTCC
Pdgf-a	Mouse	Forward	GTGCGACCTCCAACCTGA
		Reverse	GGCTCATCTCACCTCACATCT
Pdgf-b	Mouse	Forward	CGGCCTGTGACTAGAAGTCC
		Reverse	GAGCTTGAGGCGTCTTGG
Pai-1	Mouse	Forward	AGGATCGAGGTAAACGAGAGC
		Reverse	TTGGTTGAGGGAATCATTCAT
Timp3	Mouse	Forward	CACGGAAGCCTCTGAAAGTC
		Reverse	TCCCACCTCTCCACAAAGTT
TGF-β3	Human	Forward	ACTTGCACCACCTTGGACTTC
		Reverse	GGTCATCACCGTTGGCTCA
α-SMA	Human	Forward	CCCTAAAGCTTCCCAGACTTCCG
		Reverse	AGTCCCGGGGATAGGCAAA
PERIOSTIN	Human	Forward	TTGAGACGCTGGAAGGAAAT
		Reverse	AGATCCGTGAAGGTGGTTTG

Supplementary Table 2 Differentially expressed genes in IRX2-overexpressed mouse cardiac fibroblasts

Gene	*Irx2*_1	*Irx2*_2	*Irx2*_3	*Gfp*_1	*Gfp*_2	*Gfp*_3	FC (*Irx2* vs *Gfp*)	*P* value	padj
**Fibrotic genes**									
Tgf-β3	1425.88	1358.89	1570.59	1141.46	1249.39	807.58	1.36	0.001	0.006
Tgf-βr2	1897.76	1814.48	1690.65	1395.87	1453.55	1336.82	1.29	0.000	0.000
Pdgf-a	1084.04	1135.03	1211.12	942.21	981.67	852.82	1.24	0.001	0.007
Pdgf -B	317.69	305.04	252.05	121.58	117.36	156.31	2.21	0.000	0.000
Pdgfrα	4482.92	4252.44	3992.83	3302.81	3276.30	3641.64	1.25	0.000	0.001
Pdgfrβ	4301.78	4209.87	3874.17	3204.87	3359.43	2540.65	1.36	0.000	0.000
Bmp8α	73.38	84.34	80.74	40.53	42.79	39.76	1.94	0.000	0.002
Thbs3	117.04	111.93	131.29	52.91	39.12	78.15	2.12	0.000	0.000
Igf2	126.33	137.15	163.59	50.66	51.34	24.68	3.37	0.000	0.000
Igfbp6	852.74	838.66	872.71	200.37	193.15	167.27	4.57	0.000	0.000
Smad3	1466.75	1468.45	1374.71	944.46	1007.34	921.38	1.50	0.000	0.000
Col9a3	112732.54	106615.93	88615.93	75023.45	77222.82	62836.17	1.43	0.000	0.000
Col1a1	121.69	121.39	77.23	64.16	58.68	53.47	1.82	0.001	0.006
Col18a1	17035.28	16837.12	14996.10	10714.42	11128.41	10392.94	1.52	0.000	0.000
Col7a1	11111.62	10708.73	8320.56	5971.84	6163.84	5500.86	1.71	0.000	0.000
Col6a2	961.42	955.32	929.58	480.67	444.99	300.27	2.32	0.000	0.000
Col6a1	414.29	420.91	334.90	233.02	240.83	226.23	1.67	0.000	0.000
Col6a3	1694.33	1678.90	1352.24	947.84	900.98	1205.20	1.55	0.000	0.000
Col27a1	31.58	12.61	33.70	10.13	7.33	1.37	4.14	0.002	0.009
Col8a2	132.83	118.23	95.49	45.03	61.12	41.13	2.35	0.000	0.000
Col4a4	17.65	16.55	13.34	2.25	1.22	4.11	6.27	0.001	0.006
Col26a1	447.73	466.62	398.79	84.43	64.79	86.38	5.57	0.000	0.000
Mmp24	3204.74	3217.51	3079.40	2395.49	2482.90	2191.02	1.34	0.000	0.000
Mmp28	682.75	621.12	680.33	462.66	366.75	361.97	1.67	0.000	0.000
Mmp2	531.34	507.61	425.47	177.86	155.26	172.76	2.89	0.000	0.000
Mmp11	16.72	18.92	16.15	1.13	1.22	1.37	13.92	0.000	0.001
Mmp15	148.63	143.46	108.83	51.78	61.12	54.84	2.39	0.000	0.000
Timp3	17119.82	16223.89	12263.54	7815.74	8066.05	4846.84	2.20	0.000	0.000
Fgf1	107.75	100.10	126.38	57.41	35.45	58.96	2.20	0.000	0.000
Fgf 2	819.30	823.69	856.56	566.23	550.12	525.13	1.52	0.000	0.000
Fgf 22	68.74	71.73	61.08	18.01	15.89	10.97	4.49	0.000	0.000
Fgfr1	5289.21	5076.12	4534.15	4115.56	3759.18	4409.46	1.21	0.002	0.008
Fgfr3	155.13	146.61	153.06	49.53	52.57	54.84	2.90	0.000	0.000
Lama5	854.60	908.82	1025.06	271.29	305.62	274.22	3.28	0.000	0.000
Smoc2	983.72	1033.35	744.22	453.66	435.21	377.05	2.18	0.000	0.000

Icam2	46.45	25.22	37.91	16.89	11.00	13.71	2.63	0.002	0.010
Icam5	42.73	56.75	40.72	27.02	8.56	13.71	2.84	0.001	0.003
Pcdh12	65.02	63.85	60.38	28.14	33.01	30.16	2.07	0.001	0.003
Pcdhgc5	508.11	446.92	339.82	296.06	276.28	301.64	1.48	0.000	0.002
Cdh24	177.42	189.17	184.65	33.77	26.89	28.79	6.16	0.000	0.000
Cdh3	294.46	244.35	223.27	73.17	47.68	67.18	4.05	0.000	0.000
Itga3	151.41	160.80	133.40	51.78	48.90	47.99	3.00	0.000	0.000
Itga7	1167.64	1142.13	1117.74	363.60	426.65	303.01	3.14	0.000	0.000
Itga8	305.61	303.46	229.59	164.35	154.03	179.61	1.68	0.000	0.000
Itga2b	185.78	204.94	211.33	75.42	106.36	69.93	2.39	0.000	0.000
Itgb4	94.75	110.35	96.89	36.02	25.67	32.91	3.19	0.000	0.000
Wnt5b	70.60	107.20	76.53	9.01	2.44	4.11	16.34	0.000	0.000
Wnt9a	89.18	75.67	82.15	10.13	13.45	6.86	8.12	0.000	0.000
Wnt6	61.31	78.82	56.17	32.65	20.78	28.79	2.39	0.000	0.001
Wnt10b	27.87	40.99	42.83	4.50	1.22	1.37	15.74	0.000	0.000
Wnt7a	61.31	59.90	55.47	2.25	4.89	8.23	11.50	0.000	0.000
Wnt4	13.93	10.25	16.15	2.25	1.22	1.37	8.32	0.002	0.007
Wnt3	624.23	588.80	464.79	352.34	365.53	270.11	1.70	0.000	0.000
Wnt11	52.95	45.72	42.13	5.63	2.44	4.11	11.55	0.000	0.000
Wnt8b	361.35	329.48	350.35	47.28	46.45	79.52	6.01	0.000	0.000
Ctnnbip1	488.61	502.88	436.70	338.84	367.97	320.84	1.39	0.000	0.001
Tnfrsf9	78.96	81.97	113.04	12.38	22.00	34.28	3.99	0.000	0.000
Tnfrsf12a	8874.81	8888.73	8179.44	6484.04	6270.20	6632.01	1.34	0.000	0.000
Tnfrsf18	156.99	174.20	146.04	42.78	50.12	41.13	3.56	0.000	0.000
Tnfrsf25	66.88	51.23	56.17	23.64	20.78	16.45	2.86	0.000	0.000
Il34	296.32	282.18	251.35	178.99	202.93	156.31	1.54	0.000	0.001
Il12rb1	48.30	35.47	33.70	5.63	8.56	4.11	6.42	0.000	0.000
Irf3	1093.33	1097.20	1092.46	898.31	931.54	872.02	1.22	0.001	0.005
Irf8	183.92	227.79	183.25	127.20	102.69	135.74	1.63	0.000	0.001
Ifnlr1	58.52	51.23	51.96	23.64	23.23	28.79	2.14	0.001	0.005

Supplementary Table 3 Genes identified by a bioinformatics intersection analysis between RNA-seq and ChIP-seq

	RNA-seq			ChIP-seq_1B7		ChIP-seq_1C1	
Gene	Log FC (*Irx2* vs *Gfp*)	*P* value	padj	LogFC	*P* value	LogFC	*P* value
Gna11	0.43	1.86E-06	1.93E-05	0.90	0.017	0.92	0.017
Ano8	0.65	2.13E-07	2.70E-06	0.87	0.012	0.82	0.023
Mlf2	0.35	4.85E-05	3.66E-04	0.97	0.010	1.21	0.001
Gadd45b	0.94	1.40E-25	1.64E-23	1.39	0.000	1.35	0.000
Cby1	0.94	4.37E-21	3.45E-19	1.11	0.010	1.15	0.006
Pck2	0.64	2.48E-15	1.03E-13	1.08	0.023	1.14	0.016
Pxn	0.95	6.37E-14	2.22E-12	1.33	0.001	1.03	0.021
Asic1	0.72	6.59E-04	3.56E-03	1.17	0.004	0.99	0.021
Vegfa	0.34	3.37E-05	2.64E-04	0.76	0.018	1.02	0.001
Egr1	0.32	7.02E-04	3.75E-03	0.81	0.006	1.02	0.001
Tcf15	4.75	3.33E-05	2.61E-04	1.49	0.001	1.66	0.000
Nectin2	0.79	2.76E-14	1.02E-12	1.07	0.000	0.83	0.009
Zswim4	0.85	3.30E-11	7.99E-10	1.16	0.001	0.96	0.006
Kmt5c	0.61	6.61E-07	7.59E-06	0.82	0.011	0.78	0.018
Csnk1g2	0.31	1.00E-03	5.09E-03	1.00	0.002	0.88	0.009
Serpine2	0.35	7.32E-05	5.27E-04	1.60	0.000	1.06	0.034
Napa	0.30	1.13E-05	9.87E-05	0.95	0.004	0.99	0.002
Lsm14b	0.40	1.01E-05	8.97E-05	0.65	0.048	0.76	0.018
Klf10	0.35	2.40E-04	1.48E-03	0.81	0.013	0.75	0.031
Rnf126	0.44	7.30E-06	6.71E-05	1.08	0.001	0.87	0.011
Lrwd1	0.61	1.46E-07	1.91E-06	0.77	0.043	0.77	0.044
Bcl6b	1.93	5.99E-07	6.95E-06	0.98	0.009	1.34	0.000
Ccdc96	1.49	4.03E-06	3.92E-05	1.37	0.000	1.69	0.000
Six5	0.62	3.76E-04	2.19E-03	0.99	0.001	1.16	0.000
Lrrc8a	0.46	2.03E-03	9.34E-03	0.87	0.014	1.19	0.001
Bet1l	0.39	3.29E-04	1.95E-03	1.18	0.000	1.35	0.000
Fam212a	0.52	1.83E-03	8.53E-03	1.16	0.002	1.27	0.001
Ppp1cc	0.35	1.96E-04	1.24E-03	0.98	0.007	1.16	0.001
Mrpl55	0.37	1.71E-03	8.05E-03	1.07	0.004	0.92	0.021
Efs	1.57	1.60E-35	3.39E-33	2.56	0.000	1.91	0.000
Sft2d1	0.32	1.73E-03	8.12E-03	1.40	0.000	1.50	0.000
Rab13	0.62	2.48E-09	4.49E-08	1.10	0.003	0.82	0.041
Dusp5	0.57	3.30E-06	3.27E-05	1.53	0.000	0.78	0.046
Kcnb1	2.00	5.47E-12	1.47E-10	1.03	0.034	1.34	0.004
Ada	0.65	6.90E-07	7.89E-06	0.98	0.019	1.18	0.003

Tbcc	0.42	8.92E-04	4.60E-03	1.34	0.000	1.20	0.000
Ninj1	0.43	2.88E-05	2.30E-04	1.19	0.001	0.99	0.009
Rilpl1	0.56	1.30E-08	2.07E-07	0.94	0.019	1.35	0.000
Arrdc2	0.76	1.84E-04	1.18E-03	0.92	0.015	1.23	0.001
Fam131a	1.35	4.22E-12	1.15E-10	1.06	0.003	1.01	0.004
Cdkn2d	0.81	7.80E-14	2.69E-12	0.86	0.013	0.98	0.005
Cnp	0.58	6.89E-08	9.66E-07	41	0.81	0.77	0.041
Arsa	0.35	8.31E-04	4.34E-03	1.34	0.000	0.98	0.015
Tspan4	0.36	1.29E-06	1.39E-05	0.78	0.017	1.09	0.001
Prmt5	0.36	1.38E-05	1.19E-04	0.75	0.022	0.69	0.037
Acadvl	0.50	9.11E-06	8.19E-05	0.83	0.046	1.23	0.001
Arntl	0.53	9.08E-04	4.68E-03	1.58	0.000	1.01	0.011
Pacsin2	0.34	1.53E-04	1.00E-03	0.87	0.023	1.40	0.000
Vav2	0.37	3.40E-04	2.00E-03	1.17	0.002	0.84	0.042
Dolpp1	0.57	4.72E-05	3.57E-04	0.89	0.006	0.97	0.004
Ppm1m	0.68	1.09E-11	2.84E-10	1.25	0.002	1.31	0.002
Btg2	0.42	3.28E-08	4.88E-07	1.24	0.000	0.71	0.036
Kifc2	1.60	3.72E-08	5.50E-07	1.06	0.001	0.74	0.038
Mapkapk3	0.49	4.85E-08	7.00E-07	0.96	0.005	0.84	0.018
Nfic	0.47	1.99E-06	2.06E-05	0.76	0.047	0.85	0.024
Smarcd2	0.40	1.01E-04	6.96E-04	1.00	0.001	1.24	0.000
Col6a2	0.77	2.39E-13	7.78E-12	1.49	0.001	1.32	0.006
Snhg12	0.46	6.72E-07	7.71E-06	1.24	0.006	1.13	0.018
Tcf7	2.90	8.31E-13	2.51E-11	1.01	0.034	1.24	0.008
Ttyh2	0.64	6.82E-11	1.57E-09	1.03	0.005	1.11	0.002
Cd320	0.80	2.08E-06	2.14E-05	0.90	0.014	0.89	0.017
Nubp2	0.30	5.90E-04	3.24E-03	1.13	0.001	1.26	0.000
Tmem263	0.31	1.54E-03	7.35E-03	1.08	0.002	1.03	0.003
Slc2a4	5.03	4.67E-14	1.67E-12	0.87	0.028	1.24	0.001
Mif4gd	0.58	2.10E-04	1.32E-03	1.18	0.001	1.02	0.006
Prkcsh	0.21	8.35E-04	4.35E-03	1.21	0.000	1.22	0.000
Ruvbl2	0.24	1.56E-03	7.46E-03	1.41	0.000	1.23	0.000
Nudt18	0.52	1.79E-05	1.50E-04	1.09	0.001	1.02	0.002
Cisd3	0.63	4.14E-06	4.02E-05	1.34	0.000	1.02	0.006
Rab11fip4	1.98	3.72E-04	2.17E-03	1.61	0.001	1.24	0.016
