# Supplementary material for: IRX2 regulates angiotensin II-induced cardiac fibrosis by transcriptionally activating EGR1 in male mice
Source: Nat Commun. 2023 Aug 16;14:4967. doi: 10.1038/s41467-023-40639-6 (PMC10432509; doi:10.1038/s41467-023-40639-6)
Supplement: Supplementary file 3 — Reporting Summary [file 41467_2023_40639_MOESM3_ESM.pdf]

nature portfolio

Corresponding author(s): Qi-Zhu Tang
Last updated by author(s): Jul 1, 2023

Reporting Summary

Nature Portfolio wishes to improve the reproducibility of the work that we publish. This form provides structure for consistency and transparency in reporting. For further information on Nature Portfolio policies, see our Editorial Policies and the Editorial Policy Checklist.

Statistics

For all statistical analyses, confirm that the following items are present in the figure legend, table legend, main text, or Methods section.

n/a	Confirmed	
☐	☒	The exact sample size (*n*) for each experimental group/condition, given as a discrete number and unit of measurement
☐	☒	A statement on whether measurements were taken from distinct samples or whether the same sample was measured repeatedly
☐	☒	The statistical test(s) used AND whether they are one- or two-sided<br>*Only common tests should be described solely by name; describe more complex techniques in the Methods section.*
☐	☒	A description of all covariates tested
☐	☒	A description of any assumptions or corrections, such as tests of normality and adjustment for multiple comparisons
☐	☒	A full description of the statistical parameters including central tendency (e.g. means) or other basic estimates (e.g. regression coefficient) AND variation (e.g. standard deviation) or associated estimates of uncertainty (e.g. confidence intervals)
☐	☒	For null hypothesis testing, the test statistic (e.g. *F*, *t*, *r*) with confidence intervals, effect sizes, degrees of freedom and *P* value noted<br>*Give P values as exact values whenever suitable.*
☒	☐	For Bayesian analysis, information on the choice of priors and Markov chain Monte Carlo settings
☒	☐	For hierarchical and complex designs, identification of the appropriate level for tests and full reporting of outcomes
☒	☐	Estimates of effect sizes (e.g. Cohen's *d*, Pearson's *r*), indicating how they were calculated

Our web collection on statistics for biologists contains articles on many of the points above.

Software and code

Policy information about availability of computer code

Data collection	a Vevo® 3100 high resolution Preclinical Imaging 496 System (FUJIFILM VisualSonics, Toronto, Canada), NanoPhotometer® spectrophotometer (IMPLEN, CA, USA), Qubit® RNA Assay Kit on a Qubit® 2.0 Fluorometer (Life Technologies, CA, USA), NEBNext® UltraTM RNA Library Prep Kit Illumina® (NEB, USA), TruSeq PE Cluster Kit v3-cBot-HS (Illumina)
Data analysis	Image-Pro Plus 6.0 (Media Cybernetics, Silver Springs, MD, USA), Image J software (version 6.0, Bio-Rad, Hercules, CA, USA), SPSS software (version 26.0, SPSS Inc., Chicago, IL), STAR software (version 2.5.3a), MACS2 (version 2.2.7.1)

For manuscripts utilizing custom algorithms or software that are central to the research but not yet described in published literature, software must be made available to editors and reviewers. We strongly encourage code deposition in a community repository (e.g. GitHub). See the Nature Portfolio guidelines for submitting code & software for further information.

Data

Policy information about availability of data

All manuscripts must include a data availability statement. This statement should provide the following information, where applicable:

- Accession codes, unique identifiers, or web links for publicly available datasets
- A description of any restrictions on data availability
- For clinical datasets or third party data, please ensure that the statement adheres to our policy

The data that support the main findings are available within the main text and the supplementary, and Source data. The datasets generated for the RNA-seq are

available through the Gene Expression Omnibus (https://www.ncbi.nlm.nih.gov/geo/query/acc.cgi?acc=GSE236454). The datasets generated for the ChIP-seq are available through the Gene Expression Omnibus (https://www.ncbi.nlm.nih.gov/geo/query/acc.cgi?acc=GSE236455). Source data are provided with this paper. Clean data of ChIP-seq were compared to a human genome (hg19) using STAR software (version 2.5.3a). Human genome (hg19) can be downloaded from ftp://ftp.ensembl.org/pub/release-99/fasta/homo_sapiens.

Research involving human participants, their data, or biological material

Policy information about studies with human participants or human data. See also policy information about sex, gender (identity/presentation), and sexual orientation and race, ethnicity and racism.

Reporting on sex and gender	Human fibrotic heart samples were taken from the left ventricle of DCM patients undergoing cardiac transplantation. Control heart samples were obtained from normal heart donors whose hearts were not suitable for transplantation for non-cardiac reasons. These heart samples were taken independent of sex and gender. For donor group, 2 females and 4 males were included. For DCM group, 2 females and 5 males were included.
Reporting on race, ethnicity, or other socially relevant groupings	Human fibrotic heart samples were taken from the left ventricle of DCM patients undergoing cardiac transplantation. Control heart samples were obtained from normal heart donors whose hearts were not suitable for transplantation for non-cardiac reasons. These heart samples were taken independent of sex and gender. They are Chinese.
Population characteristics	Human heart samples have been used in previous reports. Human fibrotic heart samples were taken from the left ventricle of DCM patients undergoing cardiac transplantation. Control heart samples were obtained from normal heart donors whose hearts were not suitable for transplantation for non-cardiac reasons. Detailed information for the donors and patients with DCM was previously presented. All donors provided signed informed consent, and all human experiments were conducted in accordance with the Declaration of Helsinki and approved by the Renmin Hospital of Wuhan University Review Board. Age of these human research participants was between 28 and 74.
Recruitment	Patients were recruited according to their diagnosis. Recruitment was independent of sex and age. No self-selection bias or other biases existed.
Ethics oversight	All donors and patients with DCM have signed written informed consent, and all human experiments were conducted in accordance with the Declaration of Helsinki and approved by our Institutional Review Board (Renmin Hospital of Wuhan University Review Board).

Note that full information on the approval of the study protocol must also be provided in the manuscript.

Field-specific reporting

Please select the one below that is the best fit for your research. If you are not sure, read the appropriate sections before making your selection.

☒ Life sciences ☐ Behavioural & social sciences ☐ Ecological, evolutionary & environmental sciences

For a reference copy of the document with all sections, see nature.com/documents/nr-reporting-summary-flat.pdf

Life sciences study design

All studies must disclose on these points even when the disclosure is negative.

Sample size	The sample size of the animals was determined according to our previous study [PMID: 26845648]. The group sizes of the in vivo experiments were estimated based on power analysis of HW/TL with an α error of 5% and a power of 80%. To detect a 10% change in HW/TL with an expected SD of 5%, we needed 5 animals per group. In our study, sample numbers obviously fulfilled the requirement.
Data exclusions	No data were excluded from analysis.
Replication	All experiments were performed with at least three independent biological samples. All attempts at replication were successful.
Randomization	All mice were randomly assigned to specific treatment groups.
Blinding	Investigators were blind to the group allocation, animal treatment, data collection and analysis.

Reporting for specific materials, systems and methods

We require information from authors about some types of materials, experimental systems and methods used in many studies. Here, indicate whether each material, system or method listed is relevant to your study. If you are not sure if a list item applies to your research, read the appropriate section before selecting a response.

Materials & experimental systems

n/a	Involved in the study
☐	☒ Antibodies
☐	☒ Eukaryotic cell lines
☒	☐ Palaeontology and archaeology
☐	☒ Animals and other organisms
☒	☐ Clinical data
☒	☐ Dual use research of concern
☒	☐ Plants

Methods

n/a	Involved in the study
☐	☒ ChIP-seq
☒	☐ Flow cytometry
☒	☐ MRI-based neuroimaging

Antibodies

Antibodies used

anti-IRX2 (1:500, #AF0552, Affinity Biosciences), anti- CTGF (1:500, #ab6992, Abcam), anti-EGR1 (1:1000, #55117-1-AP, Proteintech, Manchester, UK), anti-SMAD3 (1:500, #9513S, Cell Signaling Technology, Danvers, MA, USA), anti-p-SMAD3 (1:500, #8769, Cell Signaling Technology), anti-POSTN (1:200, # 66491-1-Ig, Proteintech Group, Inc, Chicago, USA), anti-GAPDH (1:2000, #2118, Cell Signaling Technology), Alexa Fluor 568-conjugated goat anti-rabbit IgG (H+L) cross-adsorbed secondary antibody (1:500, #A-11011, Invitrogen), and Alexa Fluor 488-conjugated goat anti-rabbit IgG (H+L) highly cross-adsorbed secondary antibody (1:500, #A-11034, Invitrogen).

Validation

anti-IRX2 (1:500, #AF0552, Affinity Biosciences) was validated by knockout or knockdown.
anti- CTGF (1:500, #ab6992, Abcam) was validated by WB on manufacture's website and positive control.
anti-EGR1 (1:1000, #55117-1-AP, Proteintech, Manchester, UK) was validated by knockout or knockdown.
anti-SMAD3 (1:500, #9513S, Cell Signaling Technology, Danvers, MA, USA), anti-p-SMAD3 (1:500, #8769, Cell Signaling Technology) were validated by WB on manufacture's website and other researchers.
anti-POSTN (1:200, # 66491-1-Ig, Proteintech Group, Inc, Chicago, USA) was validated by knockout or knockdown.
anti-GAPDH (1:2000, #2118, Cell Signaling Technology) had a single band, and were validated by WB on manufacture's website and other researchers.
Alexa Fluor 568-conjugated goat anti-rabbit IgG (H+L) cross-adsorbed secondary antibody (1:500, #A-11011, Invitrogen), and Alexa Fluor 488-conjugated goat anti-rabbit IgG (H+L) highly cross-adsorbed secondary antibody (1:500, #A-11034, Invitrogen) were most used second antibodies, and were validated by a lot of other researchers.

Eukaryotic cell lines

Policy information about cell lines and Sex and Gender in Research

Cell line source(s)

Primary adult mouse CFs were isolated from adult mice. Primary human CFs were provided by Cell Applications Inc. (San Diego, CA). Both male and female are used. We only used primary cells.

Authentication

Primary cell line was confirmed by short tandem repeat DNA sequencing.

Mycoplasma contamination

All cells were negative for contamination with several viruses, bacteria, and fungi.

Commonly misidentified lines
(See ICLAC register)

We only used primary cells. None used in this study is found in the database of commonly misidentified cell lines.

Animals and other research organisms

Policy information about studies involving animals; ARRIVE guidelines recommended for reporting animal research, and Sex and Gender in Research

Laboratory animals

Tamoxifen-inducible Col1α2-CreER mice (strain #029567) and injury-inducible Postn-Cre mice (Postntm2.1(cre/Esr1*)Jmol, strain #029645) were purchased from The Jackson Laboratory (Bar Harbor, USA).
Mice carrying the α-MHC-MerCreMer construct (A1cfTg(Myh6-cre/Esr1*)1Jmk, strain #005657) were obtained from The Jackson Laboratory.
Mice with a conditional knockout allele of Irx2 (Irx2fl/fl) were acquired from Biocytogen Pharmaceuticals (Beijing, China). Six to eight-week-old Irx2fl/fl mice were used in our study.
These mice were free access to water and food with five mice per cage. They were kept in a specific pathogen-free (SPF) condition with 20-25°C temperature and 45-55% humidity on a regular 12 hour light/dark cycle.
Mice were sacrificed by cervical dislocation at the end of these experiments.

Wild animals

No wild animals were used in this study.

Reporting on sex

All animal experiments were performed in male mice to avoid possible shielding of estrogens.

Field-collected samples

No field-collected samples were used in this study

Ethics oversight

All animal studies were conducted in accordance with the guidelines for the Care and Use of Laboratory Animals published by the

Ethics oversight	United States National Institutes of Health (NIH Publication, revised 2011) and the guidelines of the Animal Care and Use Committee of Renmin Hospital of Wuhan University.

Note that full information on the approval of the study protocol must also be provided in the manuscript.

Plants

Seed stocks	*Report on the source of all seed stocks or other plant material used. If applicable, state the seed stock centre and catalogue number. If plant specimens were collected from the field, describe the collection location, date and sampling procedures.*
Novel plant genotypes	*Describe the methods by which all novel plant genotypes were produced. This includes those generated by transgenic approaches, gene editing, chemical/radiation-based mutagenesis and hybridization. For transgenic lines, describe the transformation method, the number of independent lines analyzed and the generation upon which experiments were performed. For gene-edited lines, describe the editor used, the endogenous sequence targeted for editing, the targeting guide RNA sequence (if applicable) and how the editor was applied.*
Authentication	*Describe any authentication procedures for each seed stock used or novel genotype generated. Describe any experiments used to assess the effect of a mutation and, where applicable, how potential secondary effects (e.g. second site T-DNA insertions, mosiacism, off-target gene editing) were examined.*

ChIP-seq

Data deposition

☒ Confirm that both raw and final processed data have been deposited in a public database such as GEO.

☒ Confirm that you have deposited or provided access to graph files (e.g. BED files) for the called peaks.

Data access links<br>*May remain private before publication.*	The datasets generated for the ChIP-seq are available through the Gene Expression Omnibus (https://www.ncbi.nlm.nih.gov/geo/query/acc.cgi?acc=GSE236455).
Files in database submission	GSM7548171 CFs, IRX2, #PCRP-1B7; GSM7548172 CFs, IRX2, #PCRP-1C1; GSM7548173 CFs, Input
Genome browser session<br>(e.g. UCSC)	NA

Methodology

Replicates	ChIP-seq was performed with an two anti-IRX2 antibodies (#PCRP-IRX2-1B7 and #PCRP-IRX2-1C1, Developmental Studies Hybridoma Bank).
Sequencing depth	All experiments were paired-end sequenced. The number of total reads are varies from 29.5M to 36.3M reads. The number of unique peaks varied from 29,201 to 42,118.
Antibodies	ChIP-seq was performed with two anti-IRX2 antibodies (#PCRP-IRX2-1B7 and #PCRP-IRX2-1C1, Developmental Studies Hybridoma Bank).
Peak calling parameters	Peak calling-format BAM, q value 0.01
Data quality	q value cutoff is 0.01 . FDR of 3540 peaks and 2540 peaks are under 5%.
Software	MACS2 (version 2.2.7.1)
